# *Coxiella burnetii *vascular graft infection

**DOI:** 10.1186/1471-2334-5-109

**Published:** 2005-12-07

**Authors:** Laurence Senn, Mario Franciolli, Didier Raoult, Alexandre Moulin, Ludwig Von Segesser, Thierry Calandra, Gilbert Greub

**Affiliations:** 1 Infectious Diseases Service, Department of Internal Medicine, University Hospital, Lausanne, Switzerland; 2 Bellinzona Hospital, Switzerland; 3 Unité des Rickettsies, Université de la Méditerranée, Marseille, France; 4 University Institute of Pathology, Lausanne, Switzerland; 5 Department of Cardio-vascular Surgery, University Hospital, Lausanne, Switzerland; 6 Institute of Microbiology, University of Lausanne, Bugnon 48, 1011 Lausanne, Switzerland

## Abstract

**Background:**

*Coxiella burnetii*, the causative agent of Q fever, may cause culture-negative vascular graft infections. Very few cases of *C. burnetii *infection of a vascular graft have been reported. All were diagnosed by serology.

**Case presentation:**

We report the first case of *Coxiella burnetii *vascular graft infection diagnosed by broad-range PCR and discuss the diagnostic approaches and treatment strategies of chronic *C. burnetii *infection.

**Conclusion:**

*C. burnetii *should be considered as etiological agent in patients with a vascular graft and fever, abdominal pain, and laboratory signs of inflammation, with or without exposure history. Broad-range PCR should be performed on culture-negative surgical samples in patients with suspected infection of vascular graft.

## Background

Infection of synthetic abdominal aortic grafts occurs in ≤1% of patients, with a higher risk (1.5–2%) for grafts that extend to the femoral location. Vascular graft infection may result from intra-operative contamination, local extension from infected adjacent tissue or by hematogenous seeding. The most commonly involved pathogens are *Staphylococcus aureus *(30%), *Enterobacteriaceae *(25%), coagulase-negative *Staphylococci *(12%), *Enterococci *(9%), *Pseudomonas aeruginosa *(7%) and *Streptococci *(5%)[1]. Cultures remain negative in approximately 5% of cases [1]. *C. burnetii *account for some of these culture-negative vascular graft infections. Very few cases of *C. burnetii *infection of a vascular graft have been reported [2-5]. All previously reported cases were diagnosed by serology. The confirmation of the vascular localization of *C. burnetii *infection was obtained after the serological diagnosis of chronic Q fever by culture [3] and/or DNA amplification of *C. burnetii *from vascular graft samples [3-5]. Here, we report a case of *C. burnetii *vascular graft infection diagnosed by broad-range PCR from a surgical sample of a para-prosthetic abscess which was confirmed by serology. To our knowledge, ours is the first case where the diagnosis was made by broad-range PCR and suggests that broad-range PCR should be considered in all cases of culture-negative vascular graft infections.

## Case report

A 63-year-old man presented to a regional hospital on September 8, 2003 with a 2-week history of diffuse abdominal pain and mild diarrhea, without fever. In 1988, he had received a Dacron aorto-bifemoral graft for an infra-renal aortic aneurysm. A computerized tomography (CT) of the abdomen revealed a para-prosthetic fluid collection. Blood cultures were sterile in the absence of any recent antibiotic therapy. Laboratory results showed a white blood cell count of 5.8 G/l, a CRP of 48 mg/l, no increase of liver enzymes and a normal serum creatinine level. Empirical ciprofloxacin and metronidazole therapy was initiated and abdominal pain improved.

**Figure 1:**
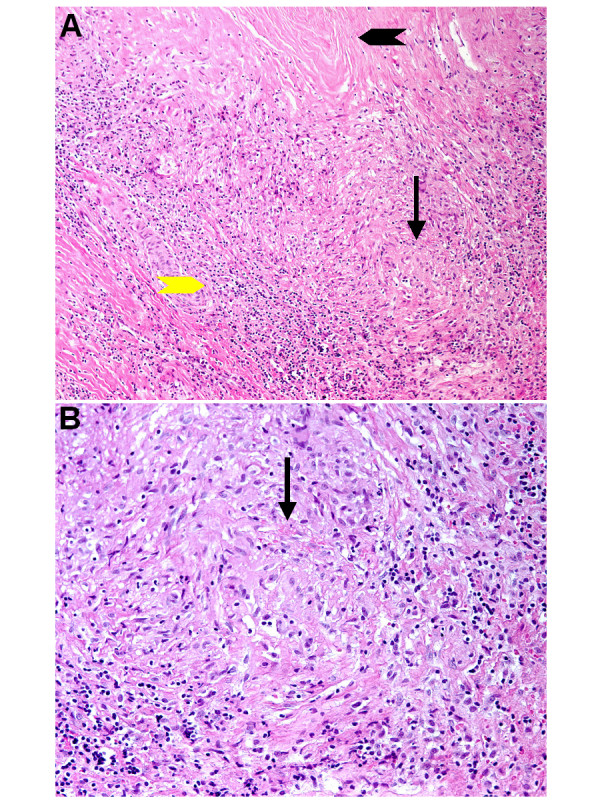
**Histology of the aortic lesion**: A. Chronic inflammatory infiltrate (yellow arrowhead), fibrosis (black arrowhead), and ill-formed granuloma (arrow). Hematoxylin-eosin, 100× magnification. B. Closer view of the ill-formed granuloma (arrow). Hematoxylin-eosin, 400× magnification.

After two months of antibiotic therapy, the patient was admitted to the University Hospital in Lausanne for removal of the vascular prosthesis because of presumed persistent infection, despite two months of antibiotic treatment. On admission, the patient was afebrile. Clinical examination was normal except for mild periumbilical tenderness on deep palpation. Laboratory results showed a normal WBC count (4.9 G/l), a normal CRP (<2 mg/l), and normal renal and liver functions. At laparotomy, extensive adhesions and a right para-iliac purulent mass were found. The prosthetic graft was partially removed, and replaced by a homograft. Multiple intra-operative specimens did not grow any microorganisms in culture. Histopathology showed a chronic inflammatory infiltrate, ill-formed non-necrotizing granulomas, and degenerative changes such as calcifications and fibrosis (Figure 1A &1B). No microorganisms could be identified using Periodic acid-Schiff, Gram, Grocott methenamine silver and Giemsa stains.

16S rRNA PCR amplification plus sequencing performed on a fragment of the para-iliac mass was positive for *Coxiella burnetii*, using the BAK11w forward and the PC3mod reverse primers [6]. The diagnosis of *C. burnetii *chronic infection was confirmed by a positive serology performed at Unité des Rickettsies, Marseille, France: phase I antibody titer (IgG): 1600, phase II antibody titer (IgG): 3200. Antibiotic therapy with doxycycline (100 mg bid orally) and chloroquine (200 mg tid orally) was started. The dose of doxycycline was increased to 300 mg daily to reach a concentration of at least 5 μg/mL [7]. Eighteen months later (May 2005), the patient was asymptomatic and serology showed persistence of high levels of phase I IgG (1600) and phase II IgG (3200).

*C. burnetii *is a strict intracellular bacterium. It is the causative agent of Q fever, a worldwide zoonosis mainly transmitted by inhalation of infected particles. There is a wide animal reservoir, and sheep and cattle are probably the main source of human infections. Our patient had no environmental exposure to *C. burnetii*. Chronic Q fever is mainly seen in patients with underlying risk factors such as valvulopathy, pregnancy, and immunosuppression. Endocarditis accounts for 73% of chronic Q fever cases, followed by vascular infection (8%), including infections of aneurysms and vascular grafts [4,8]. Given the significant morbidity and mortality of vascular infection, and given the importance of targeted and prolonged antibiotic therapy, the diagnosis of *C. burnetii *infection is crucial to a successful therapeutic outcome. We report here a rare case of *C. burnetii *vascular graft infection. This unexpected diagnosis was based on broad-range PCR. Q fever is probably underdiagnosed since the diagnosis will be missed if it is not systematically looked for in patients with vascular graft infections of unknown etiology.

The diagnosis of Q fever is most frequently made by serology. *C. burnetii *presents a variation of phase (phases I and II). Antiphase I IgG at titers of ≥1:800 by microimmunofluorescence are indicative of chronic Q fever [9,10]. The diagnosis has also be made using molecular methods including 16S rRNA PCR amplification plus sequencing or a specific *C. burnetii *PCR. To shorten the diagnostic delay, Fenollar *et al*. developed a nested-PCR assay with serum as a template which showed a sensitivity of 64% and a specificity of 100% [11]. In the present case, 16s rRNA PCR amplification plus sequencing was critical for diagnosis, since Q fever had not been suspected clinically. Broad-range PCR was also recently shown to be useful for the diagnosis of blood culture-negative infectious endocarditis, identifying an etiological agent in 35.5% of cases (11/31), including 3 cases due to *C. burnetii *[12]. The histology of Q fever endocarditis is non-specific and is characterized by fibrosis and calcifications, mild inflammation, and small or absent vegetations. Brouqui *et al*. observed a granulomatous inflammation in one third of cases with Q fever endocarditis, but well formed granuloma were not identified. In these cases, degenerative changes such as valvular calcifications or foreign body reaction prevented the establishment of a specific association between *C. burnetii *infection and granulomatous inflammation [13]. The presence of foamy macrophages, also observed in our case, may suggest a *Coxiella *infection. Definite histological diagnosis relies upon immunohistology, that may demonstrate the presence of *C. burnetii *within the cytoplasm of macrophages [13]. However, this diagnostic method is less sensitive than PCR [14]. Thus, immunohistology is unlikely to be a useful tool for the diagnosis of *C. burnetii *vascular graft infection. Cell culture is not widely available as a diagnostic technique as it requires trained technicians and a biosafety level 3 laboratory.

Tetracyclines are the antibiotics of choice for acute Q fever [10]. The bactericidal activity of doxycycline is maximal at pH 6.6. However, the *Coxiella*-containing vacuole is acidic. Therefore, addition of chloroquine that acts as an alkalinizing agent of the vacuole is essential to improve the efficacy of doxycycline therapy [15]. Combination of doxycycline and hydroxychloroquine for at least 18 months is the recommended therapy for Q fever endocarditis [15]. This combined treatment is probably also indicated in case of *C. burnetii *vascular graft infection. Since serum doxycycline concentration is correlated with decrease in levels of phase I antibodies, it is recommended to adjust the dosage of doxycycline [7]. Patients are considered cured when phase I IgG antibodies decrease below 1:800, and IgA and IgM antibodies below 1:50 [16]. In this case, after 18 months of therapy, antiphase I serology remains strongly positive suggesting persistent infection most likely due to the fact that the infected prosthesis could only be partially removed. It also shows how difficult it is to eradicate *C. burnetii *and emphasizes the need for prolonged antibiotic treatment course. Serological follow-up will guide our decision regarding the treatment duration.

## Conclusion

*C. burnetii *infection should be considered in patients with a vascular graft and unexplained low-grade fever, abdominal discomfort, laboratory signs of inflammation, and/or a history of environmental exposure. Broad-range PCR should be performed on surgical samples in patients with suspected infection of aneurysm or vascular graft. *C. burnetii *serology and/or specific *C. burnetii *PCR should also systematically be performed in cases of culture-negative vascular graft infection.

## Competing interests

The author(s) declare that they have no competing interests.

## Authors' contributions

LS, MF, DR, LVS, TC and GG were involved in patient care. LS wrote the first draft of the manuscript. AM did the histology and provided the images. All authors improved the manuscript and approved its final version.

## Pre-publication history

The pre-publication history for this paper can be accessed here:


